# Modular Functional-Metabolic Coupling Alterations of Frontoparietal Network in Schizophrenia Patients

**DOI:** 10.3389/fnins.2019.00040

**Published:** 2019-02-06

**Authors:** Qiong Xiang, Jiale Xu, Yingchan Wang, Tianyi Chen, Jinhong Wang, Kaiming Zhuo, Xiaoyun Guo, Kristina Zeljic, Wenli Li, Yu Sun, Zheng Wang, Yao Li, Dengtang Liu

**Affiliations:** ^1^First-Episode Schizophrenia and Early Psychosis Program, Division of Psychotic Disorders, Shanghai Mental Health Center, Shanghai Jiao Tong University School of Medicine, Shanghai, China; ^2^School of Biomedical Engineering, Shanghai Jiao Tong University, Shanghai, China; ^3^Institute for Medical Imaging Technology, Shanghai Jiao Tong University, Shanghai, China; ^4^Shanghai Hong Kou Mental Health Center, Shanghai, China; ^5^Department of Medical Imaging, Shanghai Mental Health Center, Shanghai Jiao Tong University School of Medicine, Shanghai, China; ^6^State Key Laboratory of Neuroscience, CAS Center for Excellence in Brain Science and Intelligence Technology, Institute of Neuroscience, Shanghai Institutes for Biological Sciences, Chinese Academy of Sciences, Shanghai, China; ^7^University of the Chinese Academy of Sciences, Beijing, China; ^8^Department of Biomedical Engineering, Zhejiang University, Hangzhou, China; ^9^Shanghai Key Laboratory of Psychotic Disorders, Shanghai Mental Health Center, Shanghai Jiao Tong University School of Medicine, Shanghai, China

**Keywords:** resting-state functional magnetic resonance imaging, magnetic resonance spectroscopy, modular organization, schizophrenia, frontoparietal network (FPN)

## Abstract

**Background:** Brain functional dysconnectivity, as well as altered network organization, have been demonstrated to occur in schizophrenia. Brain networks are increasingly understood to exhibit modular community structures, which provides advantages in robustness and functional adaptivity. The frontoparietal network (FPN) serves as an important functional module, and metabolic and functional alterations in the FPN are associated with the pathophysiology of schizophrenia. However, how intra-modular biochemical disruptions lead to inter-modular dysfunction of the FPN, remains unclear. In this study, we aim to investigate alterations in the modular functional-metabolic coupling of the FPN, in patients with schizophrenia.

**Methods:** We combined resting-state functional magnetic resonance imaging (rs-fMRI) and magnetic resonance spectroscopy (MRS) technology and acquired multimodal neuroimaging data in 20 patients with schizophrenia and 26 healthy controls. For the MRS, the dorsolateral prefrontal cortex (DLPFC) region within the FPN was explored. Metabolites including gamma aminobutyric acid (GABA), N-aspart-acetyl (NAA) and glutamate + glutamine (Glx) were quantified, using LCModel software. A graph theoretical approach was applied for functional modular parcellation. The relationship between inter/intra-modular connectivity and metabolic concentration was examined using the Pearson correlation analysis. Moreover, correlations with schizophrenia symptomatology were investigated by the Spearman correlation analysis.

**Results:** The functional topological network consisted of six modules in both subject groups, namely, the default mode, frontoparietal, central, hippocampus, occipital, and subcortical modules. Inter-modular connectivity between the frontoparietal and central modules, and the frontoparietal and the hippocampus modules was decreased in the patient group compared to the healthy controls, while the connectivity within the frontoparietal modular increased in the patient group. Moreover, a positive correlation between the frontoparietal and central module functional connectivity and the NAA in the DLPFC was found in the healthy control group (*r* = 0.614, *p* = 0.001), but not in the patient group. Significant functional dysconnectivity between the frontoparietal and limbic modules was correlated with the clinical symptoms of patients.

**Conclusions:** This study examined the links between functional connectivity and the neuronal metabolic level in the DLPFC of SCZ. Impaired functional connectivity of the frontoparietal areas in SCZ, may be partially explained by a neurochemical-functional connectivity decoupling effect. This disconnection pattern can further provide useful insights in the cognitive and perceptual impairments of schizophrenia in future studies.

## Introduction

Schizophrenia (SCZ) is a chronic and devastating psychiatric illness affecting roughly one percent of the population and typically emerging between 18 and 25 years of age. The disorder is characterized by numerous symptoms that can be broadly categorized into three groups: positive symptoms, including hallucinations, delusions, and disorganized speech and behavior; negative symptoms, including flattened affect, social withdrawal, avolition and anhedonia; and cognitive deficits, including impaired attention, working memory, executive function and verbal memory. These symptoms lead to significant impairments in everyday functioning, and impose a large societal burden (Mesholam-Gately et al., 2009). A detailed understanding of the disease mechanism is therefore critical for the development of effective treatment strategies (Sigurdsson and Duvarci, 2015). Although advances in neuroimaging technology over the past two decades have greatly improved our understanding of the relationship between structural and functional brain features with various behaviors and cognitive characteristics, the underlying neurobiological mechanism for abnormal functional connectivity in SCZ remains unclear.

Recent magnetic resonance imaging (MRI) studies of brain networks in schizophrenia have revealed a convergent trend of widespread dysconnectivity, both in terms of brain structure and function (Fornito et al., 2012; Van Den Heuvel and Fornito, 2014; Canu et al., 2015). Specifically, evidence of abnormalities in structural connectivity from DTI studies show several white matter tracts affected in chronic schizophrenia, including the bilateral superior longitudinal fasciculus, the temporal superior fasciculus, the bilateral inferior fronto-occipital fasciculus, the genu of the right internal capsule, and other fibers in the frontal and temporal areas (Fitzsimmons et al., 2013). At the same time, numerous studies report decreased cortico-cortical functional connectivity involving the temporal gyrus, the bilateral middle cingulate cortex, the right paracentral lobule, and the parietal/posterior cingulate region in schizophrenia (Liu et al., 2011; Venkataraman et al., 2012). Increasing studies have proven that neurological and psychiatric disorders are linked to the disrupted intra-network connectivity of multiple resting-state networks, such as schizophrenia and idiopathic generalized epilepsy (Liu et al., 2017; Sun et al., 2017). Hyperconnectivity in cortical regions is localized at the frontal and temporal lobes (Zhang et al., 2012). However, most studies used single MRI modality and provide limited insights into the mechanism of schizophrenia.

The frontoparietal network (FPN) has long been implicated in the cognitive control process, within which the dorsolateral prefrontal cortex (DLPFC) is one of the most important regions involved in the impaired cognitive function of patients with SCZ (Seeley et al., 2007; Cox et al., 2014). Previous studies showed the reduced functional connectivity between the bilateral DLPFC and the parietal lobe in first episode SCZ patients (Zhou et al., 2007). The FPN exhibited reduced connectivity to cerebellar networks, yet was stronger within network connectivity in SCZ patients (Repovš and Barch, 2012; Unschuld et al., 2014). However, the underlying neurochemical mechanism remain unknown and findings within the literature have been inconsistent (Repovs et al., 2011; Woodward et al., 2011). Proton magnetic resonance spectroscopy (^1^H-MRS) allows *in vivo* detection and quantification of neuronalmetabolites and neurotransmitters in the brain. Using ^1^H-MRS, alterations in the DLPFC glutamate level in patients with SCZ were shown (Marsman et al., 2013; Poels et al., 2013), while a reduction of NAA signal in the frontal lobe has been observed (Steen et al., 2005; Jessen et al., 2006). However, how intra-modular biochemical disruptions relate to the inter-modular dysfunction of the FPN remains unclear.

In this study, we aim to investigate alterations in modular functional-metabolic coupling of the FPN in patients with schizophrenia. We used a combined resting-state functional magnetic resonance imaging (rs-fMRI) and MRS approach. A graph theoretical analysis framework was utilized to investigate the functional modular architecture in schizophrenia. We hypothesize that patients with schizophrenia showed decreased FPN functional connectivity, which is associated with its intra-modular neurochemical concentration.

## Materials and Methods

### Participants

This study was conducted at the Shanghai Mental Health Center (SMHC), Shanghai Jiao tong University School of Medicine. A total of 24 patients with SCZ were recruited. All patients were in stable clinical condition and receiving antipsychotic medication. All patients met the Diagnostic and Statistical Manual of Mental Disorders, Fourth Edition (DSM-IV) (APA, 1994) diagnostic criteria for schizophrenia; the diagnosis was confirmed by a research psychiatrist using the MINI plus v 5.0 (Sheehan et al., 1998). The exclusion criteria for the study included: (1) inability to provide informed consent; (2) unstable clinical condition (e.g., aggressive and uncooperative behavior); (3) current substance abuse; (4) any other psychiatric diagnosis; (5) significant medical conditions including neurological disease, severe cardiovascular, hepatic, renal diseases; (6) pregnancy or breastfeeding; (7) previous electroconvulsive therapy.

Twenty-nine healthy volunteers were recruited from the community through media advertisements. All volunteers completed the structured clinical interview conducted by a research psychiatrist using MINI plus v 5.0 (Sheehan et al., 1998). Those with any psychiatric disorders, neurological disorders, or a positive family history of psychiatric illness were excluded.

Clinical symptoms were assessed using the Positive and Negative Syndrome Scale (PANSS)(Kay et al., 1987). Illness severity was also assessed by the Clinical Global Impressions-severity scale (CGI). This study was approved by the Institutional Review Board of the Shanghai Mental Health Center. Written informed consent was obtained from all participants.

### Image Acquisition

All MRI, MRS, and rs-fMRI data were collected on a 3.0-T Siemens Verio MR Scanner (Siemens AG, Erlangen, Germany) with a 32-channel head coil at the Shanghai Mental Health Center. During MR scanning, the participants were asked to lay supine, keep quiet and remain awake with their eyes closed. Anatomical T1-weighted images were acquired using a magnetic preparation fast gradient echo (MPRAGE) sequence with the following parameters: echo time (TE) = 3.65 ms, repetition time (TR) = 2,530 ms, field of view (FOV) = 256 × 256 mm, slice thickness = 1.0 mm and slice number = 224. The rs-fMRI data were collected with a gradient echo pulse echo planar imaging (GRE-EPI) sequence, TE = 2,000 ms, TR = 30 ms, flip angel (FA) = 90°, FOV = 22 cm, matrix = 64 × 64, slice thickness = 4 mm, slice gap = 0 mm, and slice number = 30, voxel size = 3.4 mm × 3.4 mm × 4 mm.

**Figure 1:**
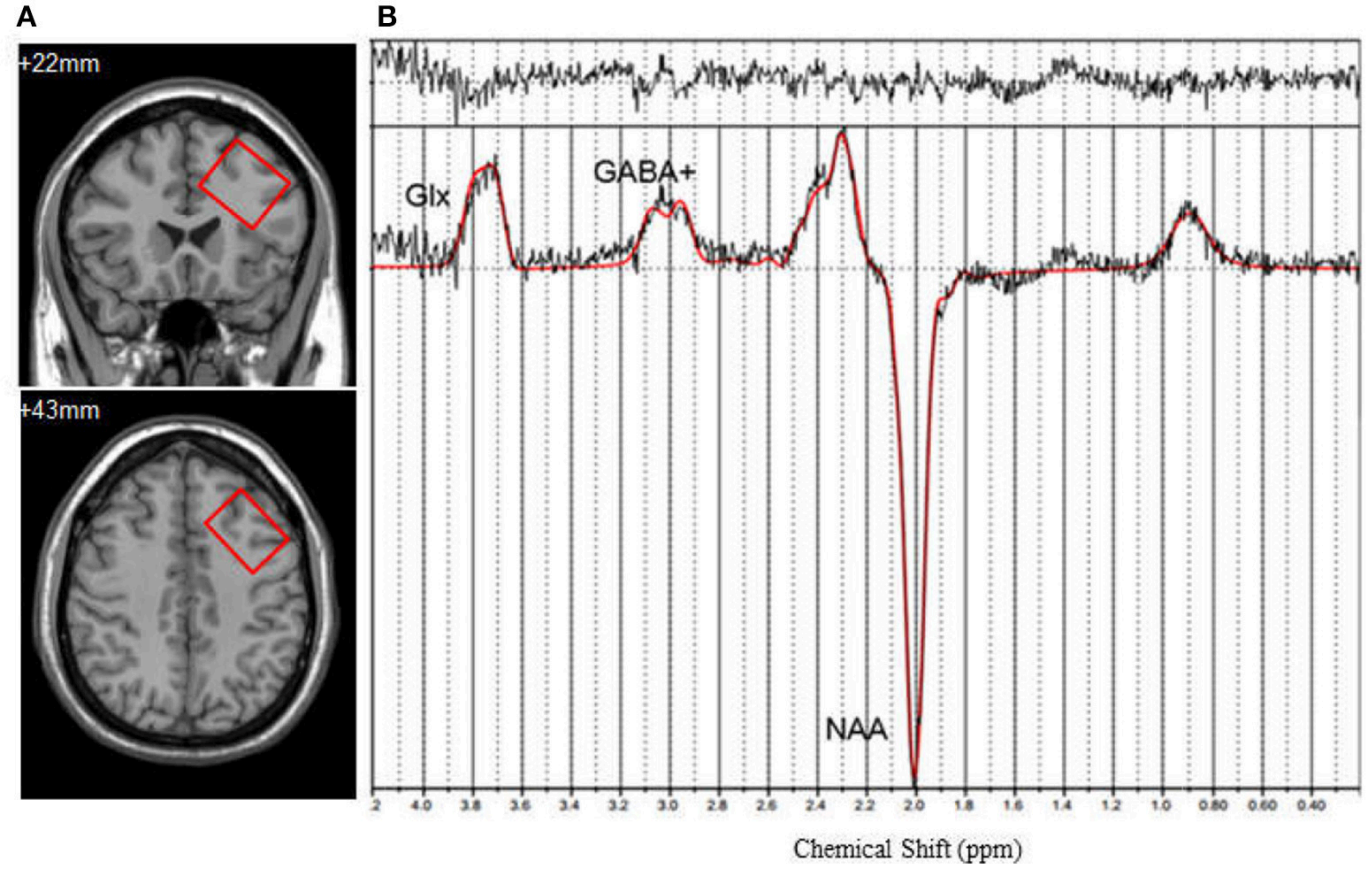
**(A)** Axialand coronal views of left dorsolateral prefrontal cortex (DLPFC) (*y* = +22, *z* = +43). Voxel size is 35 × 25 × 30 mm^3^. **(B)** ^1H^-MRS spectrum fitted by LCModel. GABA^+^, γ-Aminobutyric Acid; Glx, the compound of glutamine and glutamate; NAA, N-acetylaspartate.

For single voxel MRS data acquisition, the voxel was positioned in a consistent manner by a trained investigator as shown in Figure 1A. The DLPFC voxel upper edge was parallel to the skull in the coronal position, included the cerebral cortex, but avoided overlap with the skull, in addition to coinciding with the upper inner edge and the superior frontal sulcus. Voxel included the middle frontal gyrus. The data were acquired using a MEGA-PRESS spectral editing sequence on a Siemens system (Mescher et al., 1998). The subspectra, with or without a frequency-selective inversion pulse, were subtracted to obtain the difference spectrum to eliminate the creatine signal (Figure 1B). The following acquisition parameters were used: TR = 1,500 ms, TE = 68 ms, number of averages = 128, edit pulse frequency = 1.90 ppm, edit pulse bandwidth = 45 Hz, voxel size = 35 × 25 × 30 mm^3^, water suppression >98%.

### Functional Data Preprocessing and Network Construction

The fMRI data preprocessing was carried out using the Data Processing Assistant for Resting-State fMRI (DPARSF) (http://www.restfmri.net; Yan and Zang, 2010) software, which is based on the Statistical Parametric Mapping (SPM8) (http://www.fil.ion.ucl.ac.uk/spm) and the Resting-State fMRI Data Analysis Toolkit (REST, Song et al., 2011. http://www.restfmri.net), running under Matlab 2014a (Mathworks, USA).

After removing the first ten data volumes to avoid the influence of subject adaptation to the surroundings and magnetic equilibrium effect, slice timings and realignment were performed for the remaining data volumes. Head motion artifacts were corrected using a six parameters rigid-body transform. The individual anatomical T1-weighted images were co-registered to functional images after motion correction, using a linear transformation and were segmented into gray matter, white matter, and cerebrospinal fluid (CSF) tissue maps through a DARTEL segmentation approach. A recent study demonstrated that the global signal regression (GSR) led to major improvements in quality control benchmark correlations for simple first-order linear regression models, including the six head motion parameters, as well as mean WM/CSF signals (Parkes et al., 2017). Therefore, we regressed the nuisance covariates, including the 24 head-motion profiles (Friston 24-parameter model) (Friston et al., 1996), white matter, CSF signal, and global signal (Yan and Zang, 2010). Later on, spatial normalization was performed using the standard EPI template from the Montreal Neurological Institute (MNI) and resampled to 3 × 3 × 3 mm^3^. Subsequently, image smoothing was performed with a Gaussian kernel (FWHM = 5 mm). Linear detrending and filtering with band-passed filters (0.01–0.1 Hz) was performed to reduce the effect of very low frequency drift and high frequency physiological noise. The data were excluded if the subjects had either of the following characteristics: (1) head motion over 2.5 mm translation or over 2.5° rotation in any direction. (2) Head motion mean framewise displacement (FD) (Jenkinson) >2 × S.D. over the group mean motion.

To construct the functional connectivity matrix, we employed the automated anatomical labeling (AAL) template to parcellate the brain into 90 regions of interest, an analysis from which the cerebellum was excluded. The regional time series' were calculated in correlation and the correlation coefficient constructed the functional connectivity matrix. A Fisher's Z transformation was performed to the coefficients to ensure a normal distribution. Further, to remove weak or negative correlation, a univariate test was performed to each element of the functional correlational matrix. The results with corresponding *p-*value below the statistical threshold [*p* < 0.05, false discovery rate (FDR)corrected] were retained (Wang et al., 2013). After that, we constructed a group-averaged binary connection matrix, whose element was 1, if its corresponding correlation value was statistically significant and 0 otherwise.

### ^1^H-MRS Data Processing

The MRS data were analyzed with LCModel software (version 6.3-0D) (Provencher, 1993). The fitting was performed within the frequency spectrum ranging from 0.2 to 4.0 ppm. The quantified metabolites included γ-Aminobutyric Acid (GABA+), the compound of glutamine, and glutamate (Glx) and N-Acetylaspartate (NAA), since the signal around 3 ppm has a significant contribution from macromolecules as well as GABA. This quantified GABA concentration is often called GABA+ (O'gorman et al., 2011). For quality control, only concentrations with a Cramer-Rao lower bound < 20% were included for further analysis. Partial volume effects interfere with the quantification accuracy for MRS data (Scott et al., 2016). We performed partial volume correction for the cerebrospinal fluid (CSF) by tissue segmentation on the T1-weighted image and gray matter (GM), white matter (WM), and CSF maps registration to the spectroscopic ROI. The final absolute quantification results were corrected using the obtained fractional content (Bustillo et al., 2011).

### Modularity

Modularity is one of the most fundamental and intriguing properties of biological networks (Bullmore and Sporns, 2009). A module refers to a group of nodes that are highly connected with each other but less connected with other nodes in a network (Liang et al., 2015). Modularity reflects how well the network can be delineated into a community and the analysis aims at finding a specific partition that yields maximum modularity value (Sun et al., 2017).

The modularity value Q^W^ of a weighted network is defined as (Sun et al., 2017):

(1) $$\begin{array}{r} {\text{Q}}^{\text{W}}\left(\text{p}\right)=\frac{1}{{\text{l}}^{\text{W}}}\underset{\text{i},\text{j}\in \text{N}}{\sum }\left[{\text{w}}_{\text{ij}}-\frac{{\text{k}}_{\text{i}}^{\text{W}}{\text{k}}_{\text{j}}^{\text{W}}}{{\text{l}}^{\text{W}}}\right]{\text{δ}}_{{\text{m}}_{\text{i}},{\text{m}}_{\text{j}}} \end{array}$$

Where, N is the number of nodes in the network. l^W^ is the sum of all weights in the network. ${\text{k}}_{\text{i}}^{\text{W}}$ is the strength of node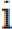. m_i_ is the module containing node[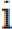]. δ_m_i_, m_j__ is equal to 1 if node [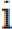] and [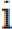] are in the same module and 0 otherwise, to ensure that only intra-modular edges are summed up. In theory, Q^W^ ranges between 0 and 1 (Newman, 2004). Q^W^ approaches 0 when the nodes are randomly partitioned or is equal to 1, if all nodes belong to the same module. Higher Q^W^ indicates significant modular structures deviating from the random network. In practice, the modularity of a network with a strong modular structure typically ranges from 0.3 to 0.7 (Newman, 2004). Modularity analysis aims to find a specific modular structure which maximizes the modularity value Q^W^.

In this study, a modified greedy optimization modular detection algorithm was adopted in a group average functional connectivity network, using the Brain Connectivity Toolbox (https://www.nitrc.org/projects/bct/). We performed a modular detection procedure on the group-averaged networks of the schizophrenia and healthy control group, separately (Meunier et al., 2009; Shin et al., 2013). In previous studies, a high sparsity threshold (including more connections) would generate a low modularity, representing a random graph. On the contrary, a low sparsity threshold (including fewer connections) would isolate the brain regions (Meunier et al., 2009). Sparsity was the number of actual edges, over the number of all possible edges. In this study, we estimated over the sparsity range of 5 to 30%, for each participant, and chose the sparsity threshold that makes the brain network of each participant fully connected with each node that is accessible to other nodes in the network. We also estimated the integrated maximum modularity over the entire sparsity range, from 5 to 30% (Supplemental Materials).

### Module Level Assessment

For module level assignment, we calculated the intra-module connectivity (*C*_*S*_) and intermodule connectivity (*C*_*S, t*_). The intra-module connectivity is defined as:

(2) $$\begin{array}{r} {\text{C}}_{\text{s}}=\frac{\sum_{\text{i},\text{jϵs}}{\text{k}}_{\text{i},\text{j}}}{{\text{l}}_{\text{s}}} \end{array}$$

where s denotes a specific module in the network; i,j are nodes in module; s,*k*_*i, j*_ is the strength between node i and j. *l*_*s*_ is the number of actual edges within modules. The inter-module connectivity (*C*_*S, t*_) is denoted as:

(3) $$\begin{array}{r} {\text{C}}_{\text{s},\text{t}}=\frac{\sum_{\text{iϵs},\text{jϵt}}{\text{k}}_{\text{i},\text{j}}}{{\text{l}}_{\text{s},\text{t}}} \end{array}$$

where s,t represents two modules in the network, respectively. i,j are nodes in module s and t. *k*_*i, j*_ is the strength between node i and j,*l*_*s, t*_ is the number of actual edges connecting to module s and module t. In order to quantitatively assess the between-group difference of modular topology and ensure comparability, we computed the intra-module connectivity and inter-module connectivity using the healthy control group modular partition as the mask. The above network metrics were calculated for each participant for further statistical analysis.

### Statistical Analysis

All the statistical analyses were performed using SPSS (version 18.0, SPSS Inc, Chicago III, USA). Two-tailed independent two sample *t*-tests were used to compare the demographic information, intra-module connectivity, inter-module connectivity and metabolite concentration between schizophrenia and healthy control groups. In addition, the relationship between network metric and metabolic concentration was examined using the Pearson correlation analysis. Their correlations with schizophrenia symptomatology, i.e., PANSS scores and its subscales, were investigated by the Spearman correlation analysis. In addition, in order to remove the impact of outliers, both the fMRI data and MRS quantification results were tested by the Outlier Detection in R software (R-3.3.3 https://www.r-project.org/).

## Results

### Demographic Characteristics and Clinical Variables

Three schizophrenia patients and three healthy controls were excluded from the study due to excessive head motion over 2.5 mm translation, or over 2.5° rotation in any direction; one schizophrenia patient was excluded due to incomplete MRS data. As a result, 20 schizophrenia patients and 26 healthy controls were included in the final analysis.

Table 1 shows the demographic and clinical characteristics of the sample. There were no significant differences in age, gender, or education between the clinical and control group. All patients were being treated with second generation antipsychotic medications (four with risperidone, nine with olanzapine, five with paliperidone, one with quetiapine, one with amisulprid). Drug choice and dosages were determined based on the treating psychiatrists' clinical judgment.

**Table 1:** Demographic and clinical characteristics of participants.

**Characteristics**	**SCZ patients (*n* = 20)**	**Healthy controls(*n* = 26)**	**Group comparison**	
	**Mean (SD)**	**Mean (SD)**	***t* or ***χ**^2^***	***p***
Age (years)	29.1 (8.0)	26.0 (4.5)	−1.606	0.115
Gender: M/F	7/13	6/7	0.580	0.446
Education (years)	13.9 (3.9)	14.5 (2.2)	0.604	0.549
Duration of illness (months)	30.02 (25.17)	\		
Episode	1.2 (0.6)	\		
PANSS-total	81.7 (12.0)			
Positive symptoms	22.4 (5.8)	\		
Negative symptoms	20.2 (5.9)	\		
General symptoms	39.2 (5.3)	\		
CGI	5 (0.88)	\		
Medication (CPZ eq mg/day)	516.05 (157.45)	\		

*SCZ, schizophrenia; PANSS, positive and negative syndrome scale, which includes positive symptoms, negative symptoms and general psychopathology subscales; CGI, clinical global impression; CPZ eq, chlorpromazine equivalence; SD, standard deviation*.

### Modular Topology of Functional Network in Schizophrenia and Healthy Controls

**Figure 2:**
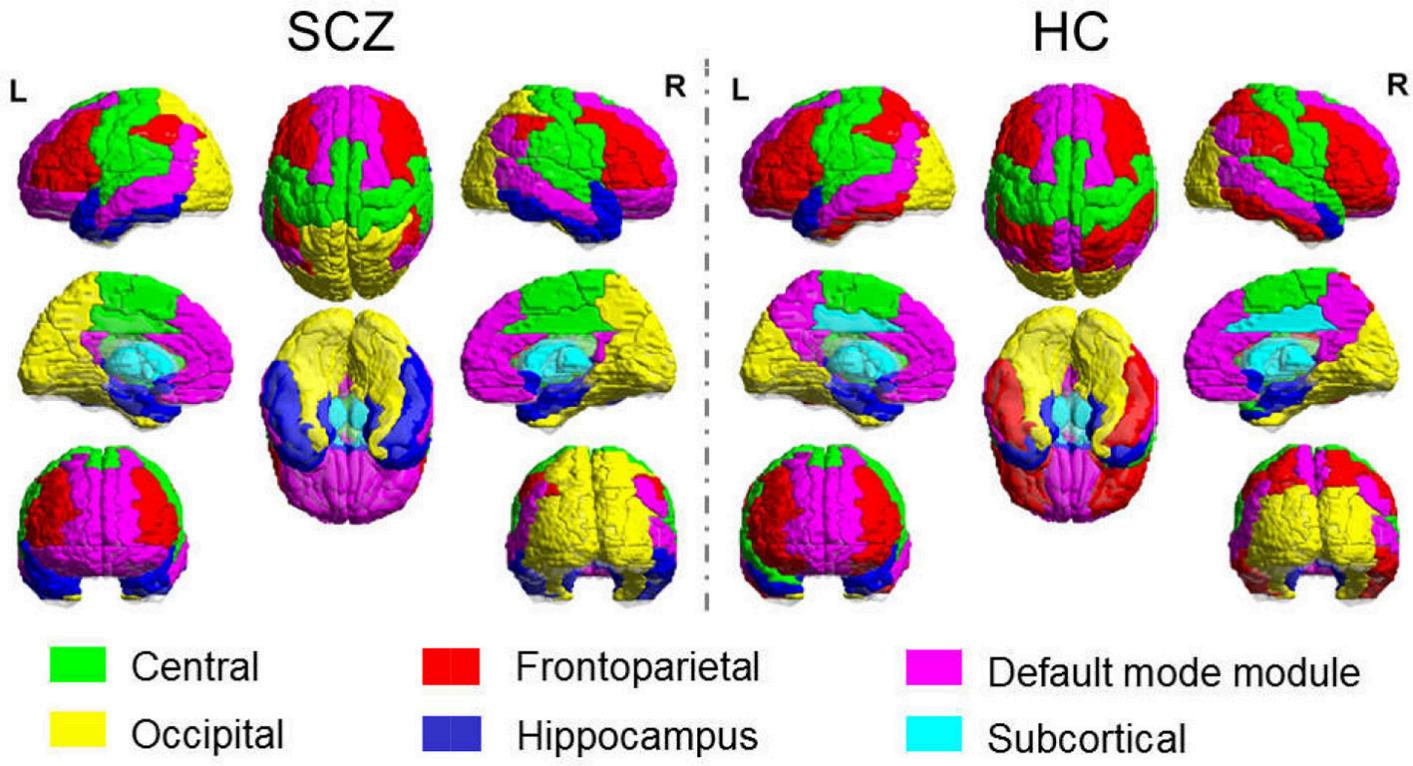
Modular Topology of Functional Network in Schizophrenia **(Left)** and Healthy Controls **(Right)**.

The sparsity threshold was selected as 15% for the modular analysis, which could capture the network backbone underlying the modular organization of the sparse feature yet remained fully-connected for each participant. The functional brain network of the healthy control comprised of six connected modules, denoted as the Central module (Module I, containing 18 regions), the Default mode module (Module II, containing 20 regions), the Frontoparietal module (Module III, containing 17 regions), the Occipital module (Module IV, containing 14 regions), the Subcortical module (Module V, containing 10 regions) and the Hippocampal module (Module VI, containing 11 regions). In the SCZ group, we also obtained six modules, with minor differences in relative size and topological profile of the brain regions. The comparison of modular topology is shown in Figure 2.

### Group Comparison of Inter/Intra-module Connectivity

Significant group differences were revealed in the inter-module functional connectivity strength from the FPN of two modules. Compared with the patient group, a significant hypo-connectivity was found between the frontoparietal module and the central module (SCZ vs. HC: 0.33 ± 0.11 vs. 0.39 ± 0.07, *p* = 0.026), between the frontoparietal module and the hippocampus module (SCZ vs. HC: 0.21 ± 0.15 vs. 0.32 ± 0.11, *p* = 0.008) in healthy controls. Significant group differences were also revealed in the intra-module functional connectivity strength for the frontoparietal module, which showed a hyper-connectivity strength for the SCZ group (SCZ vs. HC: 0.62 ± 0.14 vs. 0.49 ± 0.09, *p* = 0.001) compared to the healthy controls.

### Correlation Between Functional Connectivity and Metabolic Level

**Figure 3:**
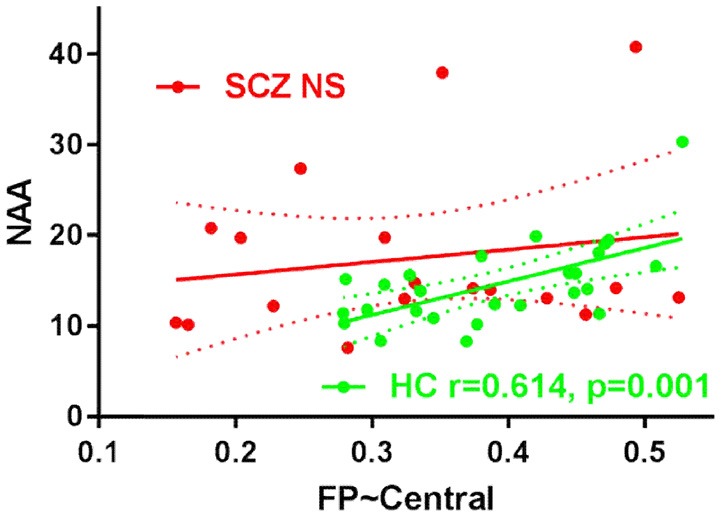
The scatter plots of the correlation FP-Central and NAA in HC group and SCZ group. NS, not significant.

Correlation analyses were performed between the intra-modular functional connectivity strength and the metabolic level in the DLPFC, including NAA, GABA+ and glutamate. A significant positive correlation was found between the FP-Central functional connectivity and the NAA in the healthy control group (*r* = 0.614, *p* = 0.001), while this relation was not found in the schizophrenia group (*r* = 0.174, *p* = 0.489) (Figure 3). No correlations were found between the FP-Central functional connectivity and the NAA in the SCZ group.

### Correlation Between Functional Connectivity and Clinical Score

**Figure 4:**
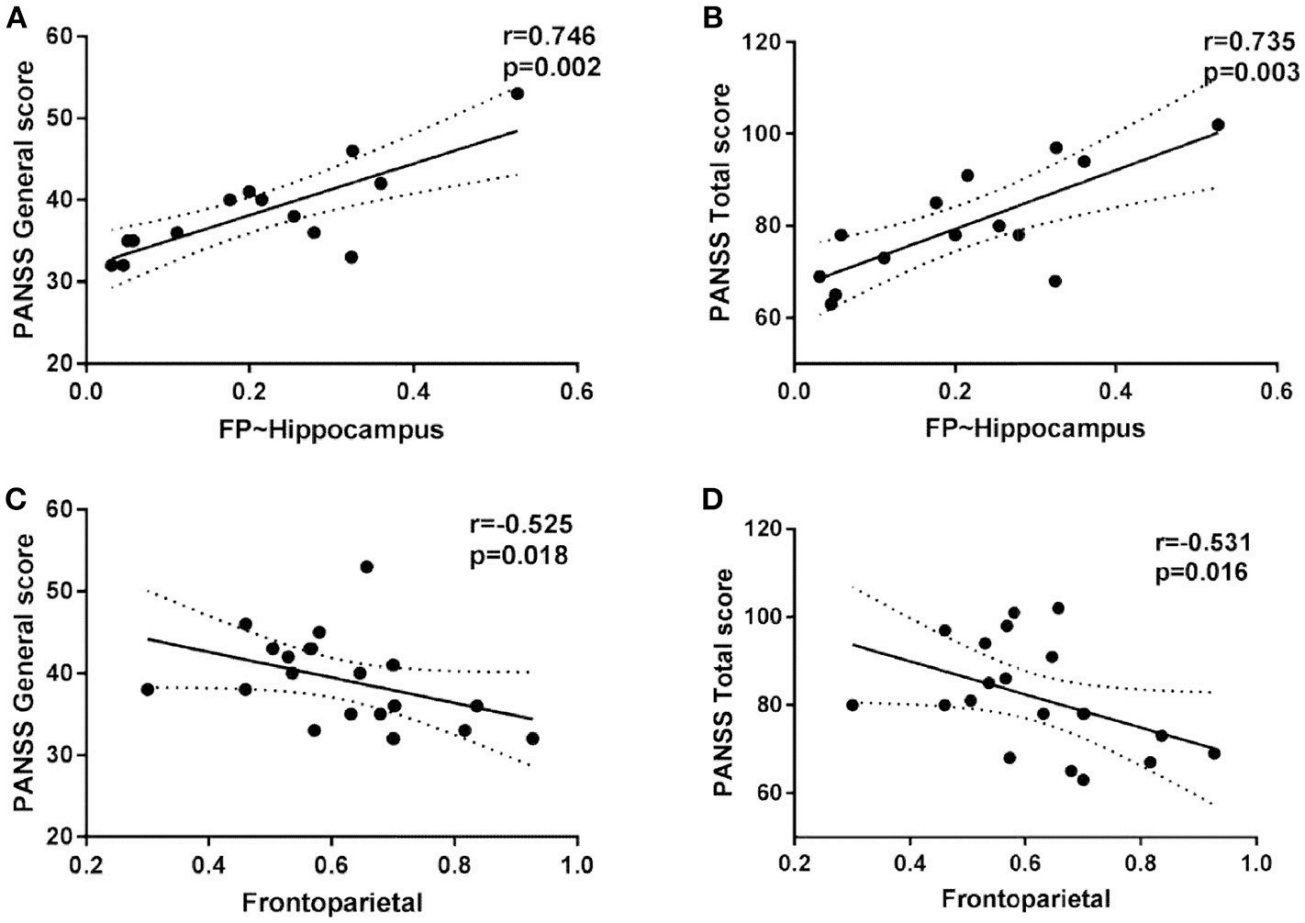
The scatter plots of the intra/inter-module connectivity and clinical symptoms in patients.

Within the SCZ group, the strength of the FP inter-module connectivity correlated negatively with the general symptoms (*r* = −0.525, *p* = 0.018) (Figure 4C) and the total score (*r* = −0.531, *p* = 0.016) (Figure 4D). The strength of the FP-Hippocampus functional connectivity correlated positively with the general symptoms (*r* = 0.746, *p* = 0.002) (Figure 4A) and the total score (*r* = 0.735, *p* = 0.003) (Figure 4B).

### Correlation Between Neuronal Metabolites in DLPFC and Clinical Features

Correlation analyses were also performed between neuronal metabolites in the DLPFC and the clinical features. A significant positive correlation was found between the GABA+ and PANSS total score (*r* = 0.700, *p* = 0.036), the NAA and the negative symptom (*r* = 0.526, *p* = 0.025), Glx, and the negative symptom (*r* = 0.636, *p* = 0.026).

## Discussion

In this study, we examined the functional-metabolic coupling alterations of the FPN in patients with schizophrenia, using multimodal neuroimaging techniques. The inter-modular connectivity between the frontoparietal and central modules as well as the frontoparietal and hippocampal modules decreased in the patient group compared to healthy controls, while the intra-modular connectivity within the frontoparietal module increased in the patient group. A significant positive correlation was found between the FP-Central functional connectivity and the DLPFC NAA level in the healthy control group but not in the SCZ group.

The present study shows significant hypo-connectivity between the frontoparietal module and the hippocampal module in the SCZ group, compared to the healthy controls. Previous studies have demonstrated that the hippocampal-prefrontal circuit plays an important role in various cognitive and emotional functions that are impaired in schizophrenia, such as attention, working memory, executive function and verbal memory, which are increasingly seen as critical for everyday functioning (Heckers et al., 1998; Meyer-Lindenberg et al., 2005; Benetti et al., 2009; Henseler et al., 2010; Rasetti et al., 2011). The decreased fractional amplitude of low-frequency fluctuations (fALFF) of the hippocampus and frontal gyrus, has also been reported in major depressive disorders (Liu et al., 2013). Different lines of evidence from structural and functional MRI have shown disturbed connectivity from the prefrontal areas to remote regions, particularly the hippocampus in SCZ. Trajectories, including the fornix, that connect the prefrontal cortex (PFC) to the hippocampus are also disrupted by many risk factors, which may promote psychosis (Zhou et al., 2008). Reduced effective connectivity and resting state functional connectivity has also been observed between the hippocampus and the mFPC (Zhou et al., 2008; Benetti et al., 2009). The results of this study suggest that connectivity between the frontoparietal module and the hippocampus module is reduced in SCZ.

The FPN showed increased intra-modular connectivity in the SCZ group compared to the healthy controls. Previous studies have shown increased functional connectivity in SCZ in the primary sensorimotor areas (SMA), including the structures of the primary sensorimotor cortex, the superior temporal gyrus (Minzenberg et al., 2009). fMRI studies of schizophrenia have also shown greater activation in these areas during various tasks (Minzenberg et al., 2009). What's more, a multivariate classification study has also demonstrated that the functional connectivity of sensor-motor network and default mode network has good diagnostic potential for social anxiety disorder (Liu et al., 2015). Furthermore, resting state fMRI studies have found increases in functional connectivity between the DLPFC and posterior or sensory cortex and the post central gyrus (Skudlarski et al., 2010; Cole et al., 2011). Our findings are consistent with the above studies, showing increased functional connectivity within the FPN and decreased connectivity to different modules, thus representing a decrease of segregation between these networks.

N-acetylaspartate (NAA), a neuron-specific brain metabolite synthesized from aspartic acid and acetyl-coenzyme A, is the most abundant signal in brain ^1^H-MRS spectra and is considered as a marker of neuronal integrity. Previous studies in schizophrenia have demonstrated correlations between a reduced NAA level and deficits in the cognitive performance of patients, including verbal learning and working memory (Bertolino et al., 2003; Ohrmann et al., 2007). About 5–10% reductions in NAA/Cr in the frontal cortex and hippocampus were found and confirmed by meta-analysis (Steen et al., 2005; Brugger et al., 2011). In the present study we found a regionally positive relationship between the NAA in DLPFC and negative symptoms in patients with schizophrenia. This is at odds with one previous study in which a regionally selective negative correlation between the prefrontal NAA-creatine ratio and negative symptom ratings in patients with schizophrenia was found (Callicott et al., 2000). Meanwhile it was implicated that treatment with antipsychotics, both typical and atypical, increases NAA measures selectively in the DLPFC of patients with schizophrenia (Bertolino et al., 2001). Although the neurobiological mechanism responsible for these changes is unclear, our findings on its correlation with the FPN inter-module connectivity, adds insight to the coupled functional-metabolic features.

Potential limitations of the study include the effects of antipsychotic medication on the neuroimaging measures. In our study, all patients were receiving antipsychotic treatment for a timeframe ranging between 1 month and over 2 years, which might add confounds to our results. Secondly, we only examined the left DLPFC, given the time constraints of scanning, which could not reflect the overall metabolic level of the frontoparietal module.

In conclusion, the present investigation examines the links between the functional connectivity and the neuronal metabolic level in the DLPFC of SCZ. Impaired functional connectivity of the frontoparietal areas in SCZ may be partially explained by a neurochemical-functional connectivity decoupling effect. This disconnection pattern can further provide useful insights in the cognitive and perceptual impairments of schizophrenia in future studies.

## Author Contributions

DL and YL conceived and designed the experiments. QX, YW, TC, KaZ, and XG contributed collecting samples and clinical assessment. QX, JX, KrZ, DL, and YL wrote the paper. YL and ZW analyzed the data. JX, WL, YS, and JW contributed imaging acquisition. All authors read and approved the final manuscript.

### Conflict of Interest Statement

The authors declare that the research was conducted in the absence of any commercial or financial relationships that could be construed as a potential conflict of interest.
