# Supplementary material for: Modular Functional-Metabolic Coupling Alterations of Frontoparietal Network in Schizophrenia Patients
Source: Front Neurosci. 2019 Feb 6;13:40. doi: 10.3389/fnins.2019.00040 (PMC6372554; doi:10.3389/fnins.2019.00040)
Supplement: Supplementary file 1 [file Table_1.docx]

Supplemental materials

## The Integrated Maximum Modularity

In the current study, modularity of functional brain network for each participant was initially estimated over a sparsity range of 5 –30%. We then chose the sparsity threshold that forms the brain network of each participant, that are fully connected with each node accessible to other nodes in the network. Finally, the sparsity threshold was set to 15%.

In order to reduce the dependency of any significant differences in the modularity (Q^W^) on the arbitrary choice of a single threshold, an integrated network metric was estimated for the modularity over sparsity range from 5% to 30%. Mathematically, the integrated modularity corresponds to the areas under the curve (AUC). Subsequently, an independent two sample t-test was conducted on the integrated values to inspect the presence of a between-group difference.

No group difference was found in the integrated modularity over sparsity range from 5% to 30% (SCZ: 22.16±0.37, HC: 22.16±0.17, t =0.048, p=0.962).

## The Alteration of the Connections Among Modules.

Left is the SCZ group, while right is the HC group.


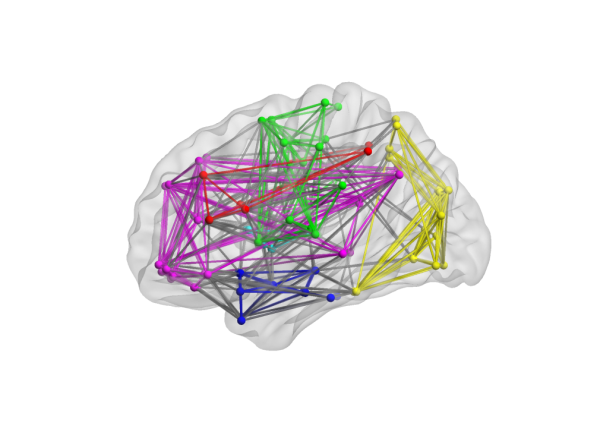

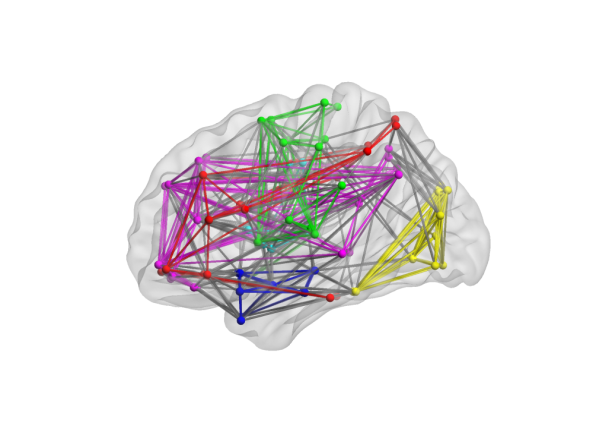


- **The Figure of response to reviewer 1:**


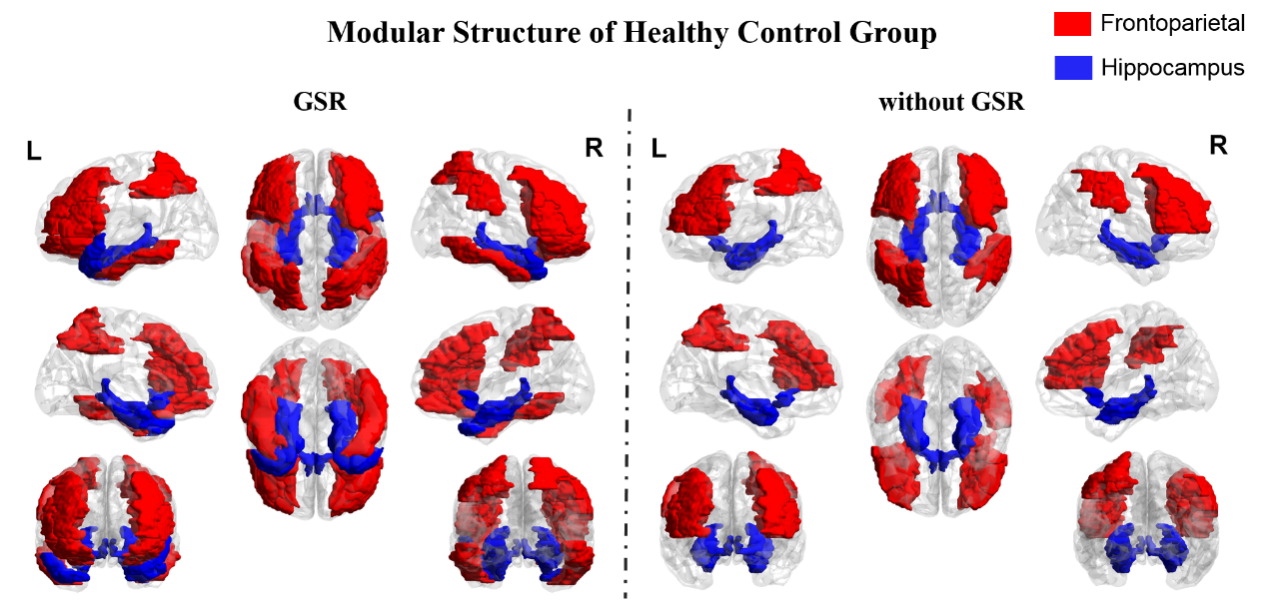


Fig 1 Comparison between the modular structure with or without global signal regression
